# Generation of glioblastoma in mice engrafted with human cytomegalovirus-infected astrocytes

**DOI:** 10.1038/s41417-024-00767-7

**Published:** 2024-03-29

**Authors:** Joris Guyon, Sandy Haidar Ahmad, Ranim El Baba, Mégane Le Quang, Andreas Bikfalvi, Thomas Daubon, Georges Herbein

**Affiliations:** 1 University of Bordeaux, INSERM U1312, BRIC, Bordeaux, France; 2 CHU Bordeaux, Department of Medical Pharmacology, Bordeaux, France; 3 University of Franche-Comté, Pathogens & Inflammation/EPILAB Laboratory, EA 4266 Besançon, France; 4 Pathology Department, University Hospital of Bordeaux, Bordeaux, France; 5 University of Bordeaux, CNRS, IBGC UMR5095, Bordeaux, France; 6 CHU Besançon, Department of Virology, Besançon, France

**Keywords:** Cancer, Cell biology

## Abstract

Mounting evidence is identifying human cytomegalovirus (HCMV) as a potential oncogenic virus. HCMV has been detected in glioblastoma multiforme (GB). Herewith, we present the first experimental evidence for the generation of *CMV-Elicited Glioblastoma Cells* (CEGBCs) possessing glioblastoma-like traits that lead to the formation of glioblastoma in orthotopically xenografted mice. In addition to the already reported oncogenic HCMV-DB strain, we isolated three HCMV clinical strains from GB tissues that transformed HAs toward CEGBCs and generated spheroids from CEGBCs that resulted in the appearance of glioblastoma-like tumors in xenografted mice. These tumors were nestin-positive mostly in the invasive part surrounded by GFAP-positive reactive astrocytes. The glioblastoma immunohistochemistry phenotype was confirmed by EGFR and cMet gene amplification in the tumor parallel to the detection of HCMV IE and UL69 genes and proteins. Our results fit with an HCMV-induced glioblastoma model of oncogenesis in vivo which will open the door to new therapeutic approaches and assess the anti-HCMV treatment as well as immunotherapy in fighting GB which is characterized by poor prognosis.

## Introduction

Glioblastoma (GB), the most common subtype of diffuse adult glioma, is a primary central nervous system (CNS) tumor presumed to arise from neural stem cells or their progenitors in the subventricular zone [1, 2]. There has been a recent paradigm shift, with increasing reliance on molecular information for diagnostic classification and prognostication within gliomas, as seen in the most recent World Health Organization (WHO) classification of CNS tumors [2]. Despite the molecular evolution of GB, it continues to be an incurable disease with poor survival.

Cancer etiological factors are assorted into genetic or environmental risk factors of which viruses are estimated to contribute to 15% of all cancer cases [3]. Human cytomegalovirus (HCMV) is a ubiquitous pathogen belonging to *Herpesviridae* family that is often detected in cancer patients [4]. Potential interrelation between HCMV and cancer has been explored and the oncomodulation paradigm was used to explain HCMV genome and/or antigens detection in a multitude of malignancies including breast cancer, colorectal, prostate, ovarian cancers and GB [4–11]. HCMV infects neural stem/progenitor cells, and human astrocytes [9, 12–15]. Going beyond oncomodulation, previous studies demonstrated HCMV’s ability to induce the transformation of human embryonal lung fibroblasts [16], human mammary epithelial cells (HMECs), ovarian epithelial cells and prostate epithelial cells in vitro [7, 8, 17]. The mainstay of treatment for GBs is surgery, followed by radiation and chemotherapy, especially temozolomide (TMZ), and the development of checkpoint inhibitors could open new possibilities to fight GB [18]. The detection of HCMV in GB biopsies could suggest the use of anti-HCMV therapies including antiviral treatment and immunotherapies directed against HCMV antigens [19–21]. New therapeutic strategies are needed and innovative experimental animal models have to be developed in addition to the patient-derived xenografts (PDX) [22] to curtail GB disease.

To assess the HCMV oncogenic potential in human astrocytes (HAs) in vivo, first, HAs were infected with HCMV-GB-9477, GB-7220, and GB-6638 clinical strains previously isolated in our laboratory and with the high-risk HCMV-DB strain resulting in the transformation of HAs into CEGBCs. Second, the spheroids generated from CEGBCs were stereotactically implanted into the brains of Ragγ2C^−/−^ mice and led to glioblastoma tumor formation in vivo.

## Materials and methods

### Cell cultures

Primary human astrocytes (HAs) and human embryonic lung fibroblasts (MRC5) were cultured as described in Supplementary Materials and Methods.

### Viruses

Clinical HCMV strains, namely HCMV-DB (GenBank KT959235), GB-9477, GB-7220, and GB-6638 were isolated from patients that were hospitalized at Besançon University Hospital (France) as described previously [7, 9, 17]. Cell-free virus stocks and infections were performed as previously detailed [17]. Careful screening of our viral stocks was conducted to rule out the presence of other oncoviruses [9, 17]. Infections of HAs and MRC5 cells, quantification of viral replication, and HCMV detection were performed as described previously [9, 17] and in Supplementary Materials and Methods. Primers used are listed in Supplementary Table 1.

### Isolation and growth of CEGBCs

Upon the appearance of large cellular clusters/structures in HAs cultures that were infected with HCMV-DB, GB-9477, GB-7220 and GB-6638 isolates, clusters were gently detached, cultured in serum-free astrocytes medium (Innoprot), and maintained in culture for more than 10 months. CEGBCs were cultured as described in Supplementary Materials and Methods.

### Flow cytometry analysis

Cells (1×10^5^) were collected from uninfected HAs, HCMV-infected HAs, and CEGBCs, fixed, permeabilized, and stained as previously reported [9]. The antibodies used are provided in Supplementary Table 2.

### RNA Cross-linking Immunoprecipitation (RNA CLIP) assay

RNA CLIP assay was performed on CEGBCs and uninfected HAs as previously reported [23, 24]. qPCR analysis of EZH2 immunoprecipitated samples (IP EZH2) and negative control (IP IgG) were normalized with respect to each input and expressed as (2^(−ΔCt)^) x100 (% Input) as previously reported [25]. The antibodies used are provided in Supplementary Table 2.

### Reverse transcription quantitative polymerase chain reaction (RT-qPCR)

The detection of transcripts was assessed by RT-qPCR as detailed previously [26] and in Supplementary Materials and Methods. Primers used are listed in Supplementary Table 1.

### Confocal microscopy

Confocal microscopy of infected human astrocytes, MRC5 cells, CEGBCs, and spheroids was performed as previously detailed [9]. The antibodies used are provided in Supplementary Table 2.

### Soft agar colony formation assay

Colony formation in soft agar (Colorimetric assay, CB135; Cell Biolabs Inc., San Diego, CA) seeded with uninfected HAs or CEGBCs was performed as described previously [9].

### Spheroid formation assay

Spheroids of CEGBCs were prepared as described previously [27, 28]. A detailed description is provided in Supplementary Materials and Methods. Spheroids were also generated from P3 cells that have been extensively characterized and have a molecular profile of the mesenchymal subgroup (from the male patient, age 64; chromosomal aberrations + [Chr 7, Chr19, 20q], −[1q42-q43, Chr9, Chr10, 20p] − [PIK3R, CDKN2A/B]) [29].

### Invasion assays

The invasion assays were performed as described previously [9].

### Mice engraftment

CEGBCs-HCMV-derived spheroids GB-6638, GB-7220, GB-9447, DB and P3 spheroids were prepared 3 days before implantation by seeding 10^4^ cells in neurobasal medium with 0.4% methylcellulose in a U-bottom 96 wells plate. After anesthetizing the mice, five spheroids of 10^4^ cells for each type were stereotactically implanted 2.2 mm to the left of the bregma into the brain parenchyma of randomly chosen Ragγ2C^−/−^ mice (8–12 weeks old) to a depth of 3 mm using a Hamilton syringe fitted with a needle (Hamilton, Bonaduz, Switzerland), following a previously described procedure [30].

### Immunohistochemistry

On histological frozen sections, hematoxylin and eosin staining (HES) was used to detect glial proliferation; this was then reviewed by trained neuropathologists from the Pathology Department, University Hospital of Bordeaux, France. For immunofluorescence on histological sections, frozen sections were processed as described previously [30]. Where specified paraffin-embedded sections were deparaffinized in toluene and hydrated serially in 100%, 96%, and 70% ethanol and distilled water. Antigen demasking was performed in citrate buffer at 95 °C for 1 h. Slides were then incubated with anti-GFAP (Cell Signalling #3670), or anti-human nestin (ThermoFisher PA5-11887) antibodies overnight at 4 °C. Sections were washed three times in PBS, and secondary fluorescent antibodies were applied (anti-mouse or anti-rabbit fluorescent antibodies) (Invitrogen A21202 and A16028). All image acquisitions were analyzed using Fiji software plug-ins. Nestin and GFAP expression localizations were analyzed using an IHC profiler. IHC profiler uses the DAB signal in images and the results are expressed as a ratio of the DAB area to the total area. The staining overlap of DAPI-IE1/2 was analyzed using the plot profile plugin. The antibodies used are provided in Supplementary Table 2.

### Statistical analysis

A detailed description of the statistical tests used is provided in Supplementary Materials and Methods.

### Ethical issues

GB biopsies were provided by the Regional tumor bank (BB0033-00024 Tumorothèque Régionale de Franche-Comté). A written informed consent for participation was obtained from all patients. The study was authorized by the local ethics committees of Besançon University Hospital (Besançon, France) and the French Research Ministry (AC-2015-2496, CNIL n°1173545, NF-S-138 96900 n°F2015). Briefly, genomic DNA was isolated from patient biopsies, and HCMV presence was identified by qPCR using specific primers against the IE1 gene. Human papillomavirus (HPV) and Epstein-Barr virus (EBV) were not detected in the GB biopsies as confirmed by qPCR screening. RNA was extracted from the biopsies, and following reverse transcription the expression of EZH2, Myc, and GAPDH was assessed by real-time qPCR. Primers used are listed in Supplementary Table 1.

Male RAGγ2C^−/−^ mice were housed and treated in the animal facility of Bordeaux University (“Animalerie Mutualisée Bordeaux”). All animal procedures were performed according to the institutional guidelines and approved by the local ethics committee (agreement number: #23802).

## Results

### Glioblastoma-like spheroids obtained following the infection of HAs with four high-risk HCMV clinical isolates

**Fig. 1:**
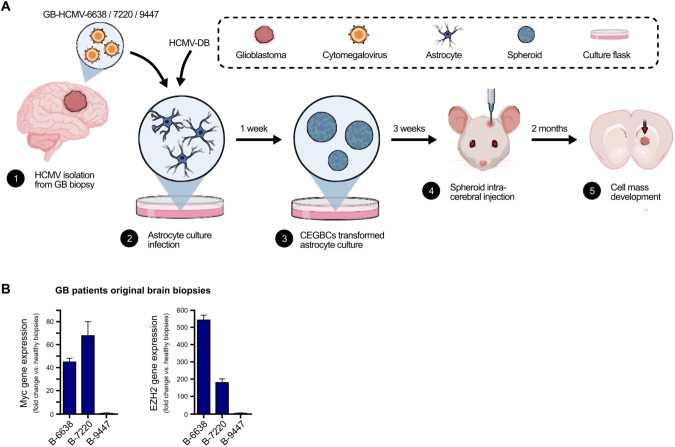
Protocol for the generation of glioblastoma tumors in mice engrafted with HCMV-infected astrocytes. **A** A scheme of the experimental model used where HAs infected with HCMV GB-6638, GB-7220, GB-9447, and DB generated CEGBCs spheroids. After three weeks, the latter were stereotactically xenografted into the brains of randomly chosen Ragγ2C^−/−^ mice generating a cell mass development day 40 to 50 post-xenografting. **B** Tumor landscape of the three (oncogenic) HCMV strains isolated from GB patients based on Myc and EZH2 expression as measured by RT-PCR.

**Fig. 2:**
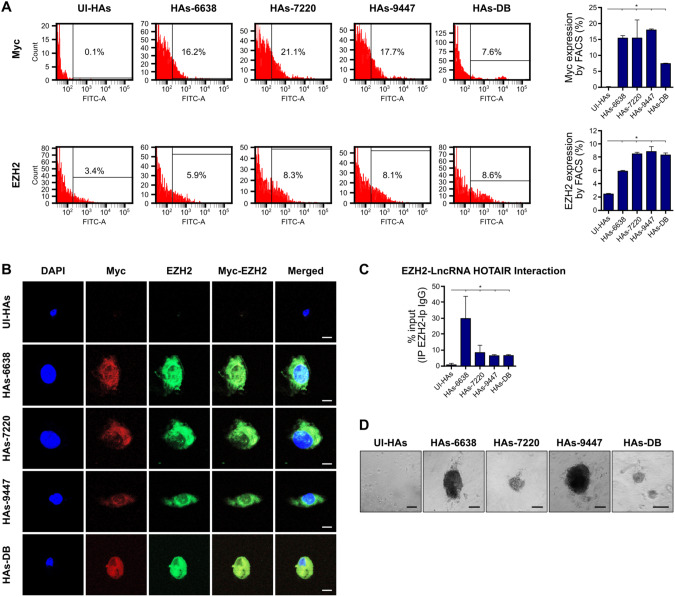
Activation of Myc-EZH2 oncogenic pathway and colony formation in soft agar in HAs infected with the GB-HCMV strains. **A** Myc and EZH2 protein expression as measured by FACS at day 3 post-infection in uninfected HAs and HAs infected with HCMV-GB and HCMV-DB strains. Histogram representing Myc and EZH2 expression in uninfected HAs and the HCMV-GB strains and HCMV-DB as measured by FACS. Data are represented as mean ± SD of two independent experiments. *p value ≤ 0.05. **B** Confocal microscopic images of Myc and EZH2 staining in HAs infected with the isolated HCMV-GB strains. Nuclei were counterstained with DAPI; magnification ×63, scale bar: 10 μm. **C** Histograms representing the lncRNA HOTAIR transcript detection in the EZH2 IP samples of HAs infected with HCMV-GB and DB strains, as measured by RT-qPCR. Mouse anti-IgG was used as an isotype control. Data are represented as mean ± SD of two independent experiments. *p value ≤ 0.05. **D** Colony formation in soft agar seeded with HCMV-GB and HCMV-DB -infected HAs; UI HAs were used as a control. Formed colonies were observed under an inverted light microscope. Magnification x200, scale bar: 100 µm.

Our goal was to assess in vivo the oncogenic potential of the HCMV GB-6638, GB-7220, and GB-9447 strains that were isolated from GB tumors (GB-6638, 7220, and 9447) including the high-risk HCMV-DB strain that was isolated from a cervical swab in our laboratory. We followed the experimental plan described in Fig. 1A. Afterward, we infected HAs with these four HCMV clinical strains and their transformation potential of HAs into *CMV-Elicited Glioblastoma Cells* (CEGBCs) was assessed in vitro. Then the derived CEGBCs spheroids were stereotactically xenografted into the brains of randomly chosen Ragγ2C^−/−^ mice to determine their tumorigenicity (Fig. 1A). Previously, we reported that among the thirty-seven GB biopsies, eleven GB biopsies were considered for HCMV isolation. The strains were isolated from MGMT promoter methylated (*n* = 4) and MGMT promoter unmethylated (*n* = 7) GB tumors by tissue disruption and filtration, and were subsequently grown in MRC5 cells showing a peak of viral load (1–3 log) around day 20 post-infection [9]. Herein, three HCMV-GB strains were selected, based on their clinical and biological properties, to assess their potential tumorigenicity in infected HAs xenografted into mice. Based on their GB gene expression profile of Myc and EZH2 and Akt GB-6638, 7220 and 9447, displayed high (Myc^High^EZH2^High^Akt^High^), intermediate (Myc^High^EZH2^Intermediate^Akt^Low^), and low (Myc^Low^EZH2^Low^Akt^Low^) aggressiveness, respectively (Fig. 1B and Suppl. Fig. 1). The MGMT promoter of GB-6638 and 7220 was unmethylated in contrast to the methylated one in GB-9447 (Supplementary Table 3). In addition to the three GB HCMV strains, we tested the high-risk oncogenic HCMV-DB strain isolated from the cervical swab of a pregnant woman [7, 31] (Fig. 1A). Myc was overexpressed in HAs infected with the three HCMV-GB strains and HCMV-DB, unlike uninfected HAs as measured by flow cytometry (*p* value = 0.04) and confocal microscopy (Fig. 2A). To a lesser extent, elevated EZH2 expression was detected in all three HCMV-GB strains and HCMV-DB, unlike uninfected HAs as measured by flow cytometry (*p* value = 0.05) and confocal microscopy (Fig. 2A, B). In agreement with the presence of the methyltransferase EZH2 in CEGBCs possessing glioblastoma-like traits obtained following chronic infection of HAs with HCMV [9], we observed the interaction of cellular lncRNA HOX antisense intergenic RNA (HOTAIR) transcripts with EZH2 using RNA CLIP assay (Fig. 2C). Cellular lncRNA HOTAIR transcript, reported as a poor prognostic factor in cancers [32, 33] was detected in the EZH2 immunoprecipitated samples corresponding to the three CEGBCs-GB strains and in CEGBCs-DB compared to uninfected HAs (*p* value = 0^.^04) (Fig. 2C). lncRNA HOTAIR interaction with EZH2 was high in GB-6638 CEGBCs, intermediate in GB-7220 CEGBCs and low in GB-9447 CEGBCs (Fig. 2C). All three HCMV-GB isolates and HCMV-DB transformed HAs as measured by soft agar colony formation assay (Fig. 2D).

**Fig. 3:**
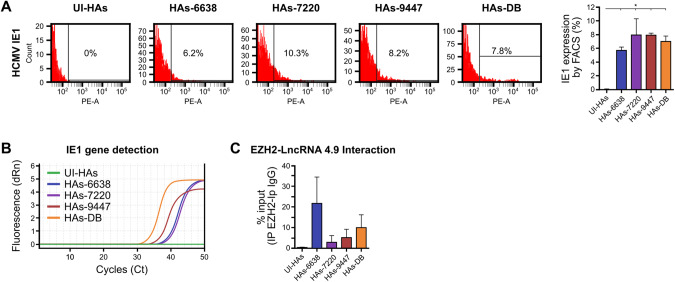
Detection of HCMV and identification of the lncRNA4.9/EZH2 complex in HAs infected with the GB-HCMV strains. **A** IE1 protein detection in HAs infected with the GB-HCMV strains and HCMV-DB as measured by FACS; UI HAs were used as a control. Histogram representing IE1 expression in uninfected HAs and the HCMV-GB strains and HCMV-DB as measured by FACS. **B** IE1 gene detection in HAs infected with the GB-HCMV strains and HCMV-DB as measured by qPCR. **C** lncRNA 4.9 transcript detection in the EZH2 IP samples of HAs infected with HCMV-GB and DB strains, as measured by RT-qPCR. Mouse anti-IgG was used as an isotype control. Data are represented as mean ± SD of two independent experiments.

Viral protein IE1 was detected in HAs infected with the three HCMV-GB strains and HCMV-DB compared to uninfected HAs using flow cytometric analysis (Fig. 3A). The IE1 gene was detected in all four samples using qPCR (Fig. 3B). In agreement with the previously reported interaction between viral lncRNA4.9 and EZH2 [23], lncRNA4.9 was detected in the EZH2 immunoprecipitated samples corresponding to the three CEGBCs-GB strains and in CEGBCs-DB compared to uninfected HAs (Fig. 3C).

**Fig. 4:**
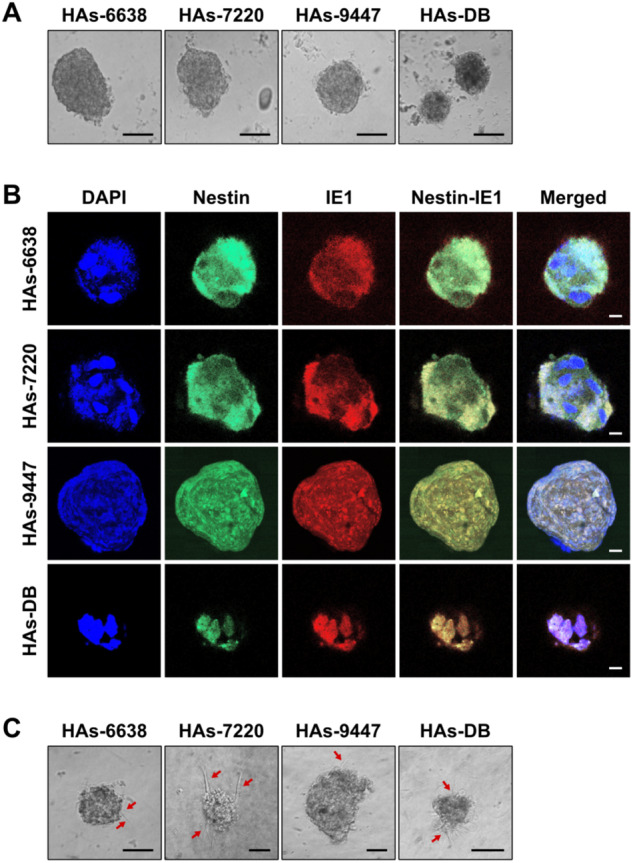
Spheroid forming and invasion potentials of the HCMV-GB and HCMV-DB strains used for xenografts. **A** Microscopic images of the spheroids generated from the isolated GB HCMV and DB strains; magnification ×100, scale bar: 20 μm. **B** Confocal microscopic images of concomitant Nestin/IE1 staining in spheroids generated from the isolated HCMV-GB and DB strains. Nuclei were counterstained with DAPI; magnification ×63, scale bar: 10 μm. **C** Microscopic images showing the invasion potential of CEBGCs-derived spheroids through protrusions (red arrows); magnification ×200, scale bar: 20 μm.

Spheroids were generated 24–48 h post-seeding the HAs infected with the clinical HCMV-GB strains and HCMV-DB (Fig. 4A). High nestin levels were detected in spheroids generated from the three HCMV-GB strains and HCMV-DB (Fig. 4B). Nestin and IE1 were concomitantly expressed in spheroids generated from all HCMV-GB strains and HCMV-DB (Fig. 4B). Further, a 3D collagen-invasion assay was performed to evaluate the invasiveness potential of the spheroids generated from HCMV-GB and HCMV-DB strains. Protrusion and invading cells were observed with the three HCMV-GB and HCMV-DB strains (Fig. 4C). Taken together, a Myc^High^ EZH2^High^ molecular profile with stemness and invasiveness was observed with all four high-risk HCMV strains in vitro, paving the way for the engraftment of these spheroids in the brain of immunosuppressed mice as reported previously with spheroids originating from patients with GB, namely PDX [34].

### Generation of glioblastoma-like tumors in mice xenografted with HCMV-high-risk spheroids

**Fig. 5:**
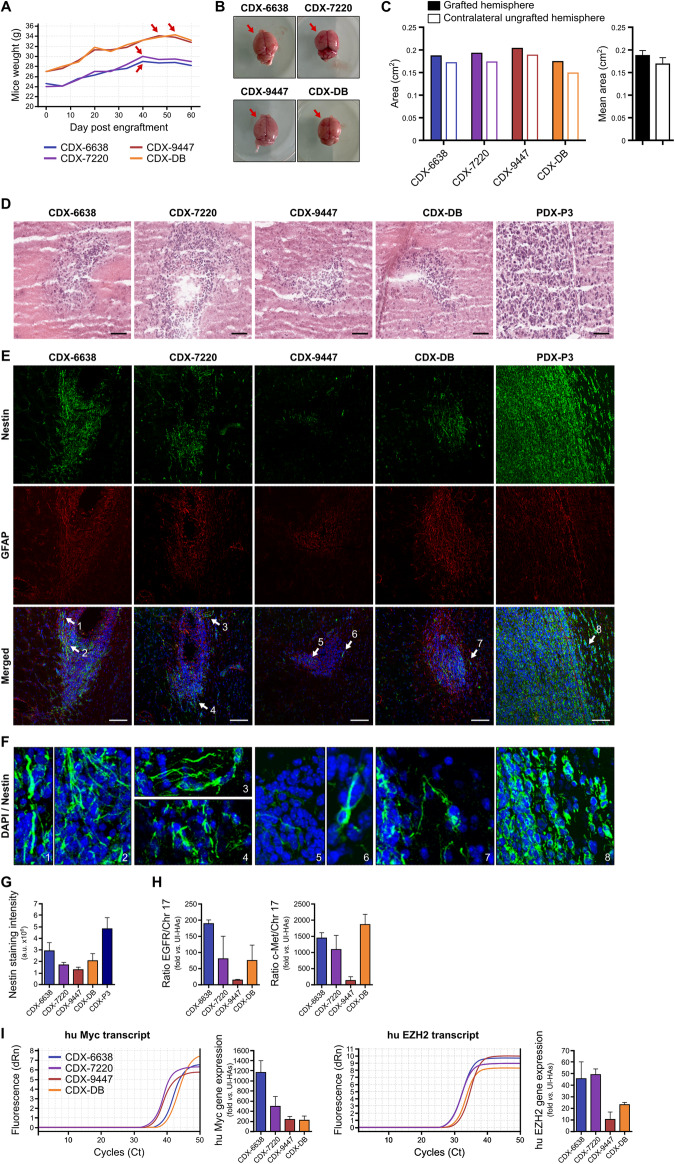
Generation of CMV-Derived Xenografts (CDX) and CDX tumors which displayed glioblastoma-like immunohistochemical and molecular phenotypes. **A** Loss of weight in mice grafted with CEGBC-derived spheroids. **B** Brain photography after mice sacrifice; the left hemisphere (red arrows) corresponds to the one that has been implanted. **C** Histogram indicates the surface area of each cerebral hemisphere: grafted left and ungrafted control right. **D** Presence of glial proliferation combining increased cellularity and nuclear atypia with enlarged hyperchromatic nuclei in engrafted hemispheres. Tissue was stained using HES. Scale bar: 50 µm. **E**–**G** Using immunohistochemistry staining, CDX tumor biopsies were positive (**E**, **F**) for nestin (CDX-6638 > CDX-7220 > CDX-9447); GFAP, a marker of reactive astrocytes upon viral infection was also detected. **F** Zoom in on the areas indicated by the arrows in (**E**) showing nestin filament proteins. Scale bar: 100 µm. **G** Histogram indicates the staining intensity of nestin of images presented in (**E**). **H** EGFR and cMet gene amplification in CDX tumor biopsies (fold increase versus normal uninfected astrocytes). **I** Human Myc and EZH2 gene expression in CDX tumor biopsies. *Left panel:* RT-qPCR curves of human Myc and EZH2 gene expression in CDX tumor biopsies. *Right panel:* Histograms represent the human Myc and EZH2 gene expression in CDX tumor biopsies (fold increase versus uninfected HAs). Data are represented as mean ± SD of three independent experiments.

With previously mentioned encouraging results, we sought to determine the oncogenic potential of CEGBCs-HCMV-derived spheroids in vivo. CEGBCs-HCMV-derived spheroids GB-6638, GB-7220, GB-9447, DB and P3 spheroids were stereotactically implanted into the brains of randomly chosen Ragγ2C^−/−^ mice. P3-derived spheroids were used as a positive control. The mice were weighed on weekly basis. A decrease in the mice’s body weight was noticed at day 40 for GB-6638 and GB-7220 and around day 50 for GB-9447 and DB (Fig. 5A, red arrows) along with the unusual behavior observed including sleepiness and anorexia; thus the mice were sacrificed. The engrafted hemispheres were slightly increased in size (approximately 0.019 cm^2^) versus their counterpart (Fig. 5B, C).

Grafts with a glioblastoma-like phenotype originating from HCMV-infected HAs/CEGBCs were termed “CMV-Derived Xenografts” or CDX similar to the previously reported patient-derived xenografts (PDX). Using HES staining, microfoci of diffuse glial proliferation combining increased cellularity and moderate nuclear atypia characterized by slightly enlarged hyperchromatic nuclei with irregular contours were present in the grafted hemisphere of mice injected with CEGBC-GB spheroids and CEGBC-DB spheroids, namely CDX-6638, CDX-7220, CDX-9447 and CDX-DB (Fig. 5D). Abundant diffuse high-grade glial proliferation with more pronounced nuclear atypia, accompanied by a few giant cells and high mitotic activity was detected in the grafted hemisphere of mice injected with P3-derived spheroids, namely PDX-P3, used as a positive control (Fig. 5D). Using immunohistochemistry staining, the tumor biopsies of mice injected with CEGBC-GB spheroids and CEGBC-DB spheroids displayed nestin-positive staining in the grafted hemisphere (Fig. 5E). Nestin staining was strong for CDX-6638, intermediate for CDX-7220 and CDX-DB and low for CDX-9447 (Fig. 5E, F). The tumor biopsies of mice injected with P3 (PDX-P3) were very strongly positive for nestin (Fig. 5E, F). GFAP staining was present in CDX-7720, CDX-6638 and CDX-9447 biopsies but mostly in the surrounding area of the nestin-positive invasive regions, indicating that the two stainings were mutually exclusive (Fig. 5E). GFAP was dimly expressed in PDX-P3 biopsies (Fig. 5E). Among the three CDX biopsies, the nestin level was the highest in the CDX-6638, intermediate in the CDX-7220 and the lowest in the CDX-9447 (Fig. 5G). The nestin level was intermediate in the CDX-DB and very high in the highly invasive PDX-P3 positive control (Fig. 5G).

The 2021 WHO Classification of CNS Tumors indicated that EGFR gene amplification is an important hallmark of glioblastoma multiforme [2]. Therefore, we assessed the level of EGFR gene amplification in the tumor biopsies. We detected EGFR gene amplification in the grafted hemisphere for CDX-7720, CDX-6638, and CDX-9447 biopsies as well as for the CDX-DB biopsy (Fig. 5H). In contrast to high levels of EGFR gene amplification in CDX-6638, CDX-7720, and CDX-DB xenografted mice with 189, 81 and 75-fold amplification, respectively, limited EGFR gene amplification was observed in CDX-9447 xenografted mice with 15-fold amplification (Fig. 5H). Similarly, although all three CDX-6638, CDX-7220 and CDX-DB displayed amplification of the cMET gene with 1448, 1099 and 1870-fold amplification, respectively, a limited cMET gene amplification was detected in CDX-9447 grafted mice with 138-fold amplification (Fig. 5H). The tumor microenvironment of CDX-grafted mice with the three HCMV GB and DB strains was assessed for Myc and EZH2 gene expression. High levels of Myc expression were detected in CDX-6638, intermediate levels in CDX-7220 and low expression in CDX-9447 (Fig. 5I). In contrast to enhanced EZH2 expression in CDX-6638 and CDX-7220, low EZH2 expression was observed in CDX-9447 CDX (Fig. 5I). Low Myc and intermediate EZH2 expression was detected in CDX-DB (Fig. 5I).

**Fig. 6:**
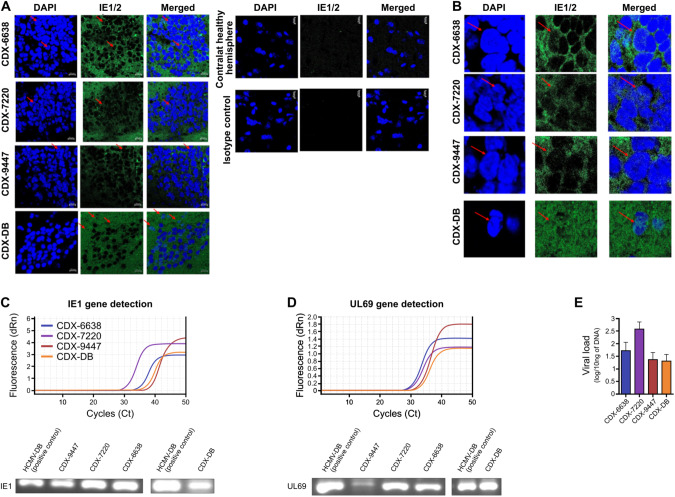
HCMV detection in CDX tumors. **A** Using immunohistochemistry staining, CDX tumor biopsies were positive for IE1/2 protein (red arrows indicated nuclear staining). Nuclei were counterstained with DAPI. IE1/2 protein detection in CDX engrafted hemisphere (tumor tissue) but not in the ungrafted contralateral hemisphere. Magnification ×63, scale bar: 10 μm. **B** IE1/2 protein detection in the nucleus and cytoplasm of cells present in the CDX tumor (red arrows indicated nuclear staining). Magnification ×63, scale bar: 10 μm. **C** IE1 gene detection (117 bp amplicon) in CDX tumors as measured by qPCR and gel loading. **D** UL69 gene detection (199 bp amplicon) in CDX tumors as measured by qPCR and gel loading. **E**. Histogram represents HCMV viral load in CDX brain; mean ± SD of four independent experiments.

Using immunohistochemistry staining, the tumor biopsies of mice injected with CEGBC-GB spheroids and CEGBC-DB spheroid displayed IE-positive staining in the grafted hemisphere (Fig. 6A). The IE protein was detected in the cytoplasm and to a lesser extent in the nucleus of CDX grafted hemispheres (Fig. 6A, B) in contrast to the contralateral ungrafted hemisphere which was IE-negative (Fig. 6A). We detected the IE1 and UL69 genes in all three CDX-7720, CDX-6638, CDX-9447 and CDX-DB by qPCR (Fig. 6C–E). We then sequenced the IE1 and UL69 amplicons isolated from CDX-grafted mice. The IE1 and UL69 amplicon sequences in CDX-6638, 7220, 9447 and DB were mostly analogous to the IE1 and UL69 genes of the HCMV-DB clinical strain (Supplementary Table 4).

### PDX-P3 harbors HCMV

**Fig. 7:**
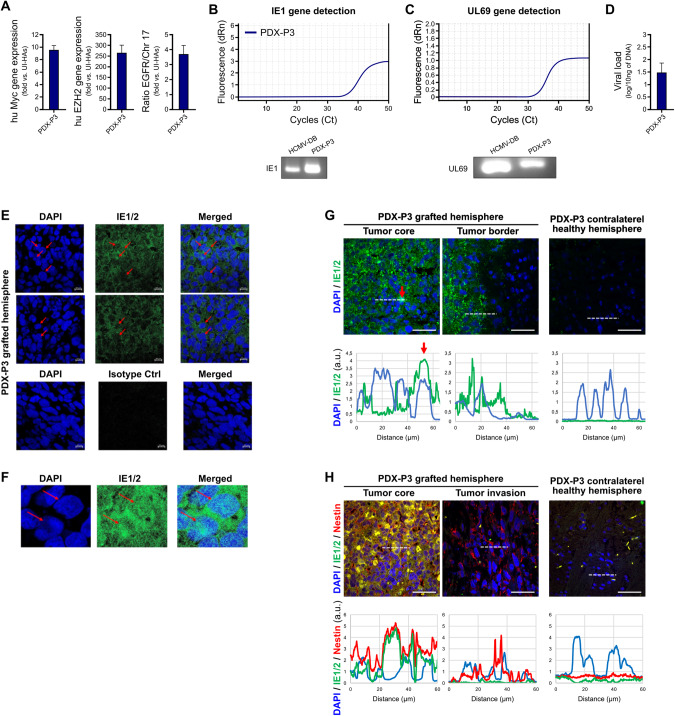
PDX-P3 harbors HCMV. **A** Enhanced Myc and EZH2 expression parallels EGFR gene amplification in PDX-P3 tumor biopsies. Data are represented as mean ± SD of three independent experiments. **B** IE1 gene detection (117 bp amplicon) in PDX-P3 tumor as measured by qPCR and gel loading. **C** UL69 gene detection (199 bp amplicon) in PDX-P3 tumor as measured by qPCR and gel loading. **D** Histogram represents HCMV viral load in PDX-P3 tumor; mean ± SD of four independent experiments. **E** Using immunohistochemistry staining, PDX-P3 tumor biopsies were positive for IE1/2 protein (red arrows indicated nuclear staining). Nuclei were counterstained with DAPI. Magnification x63, scale bar 10 μm. **F** IE1/2 protein detection in the nucleus and cytoplasm of cells present in the PDX-P3 tumor (red arrows indicated nuclear staining). Magnification x63, scale bar: 10 μm. **G** Tissue distribution of IE1/2 protein in PDX-P3. *Upper panel*: Detection of IE1/2 protein (green) and nucleus (blue) in the tumor core, in the tumor border but not in the healthy contralateral area. Nuclei were counterstained with DAPI. Scale bars: 50 µm. *Lower panel*: Graphs represent the staining intensity profile (a protein expression indicator) of IE1/2 and DAPI, delineated by the white dashed line (in the corresponding upper panel). The red arrow shows an overlap between the two staining for a cell. **H** Tissue distribution of IE/nestin in PDX-P3. *Upper panel:* IE/nestin double staining indicates the concomitant presence of the two proteins in the tumor core, but only nestin is detected in the invasive area. Nuclei were counterstained with DAPI. Scale bars: 50 µm. *Lower panel*: Graphs represent the staining intensity profile (a protein expression indicator) of IE1/2, nestin and DAPI, delineated by the white dashed line (in the corresponding upper panel).

As previously reported by other research groups [35] and specified above in Fig. 5D–G, the tumor biopsies of mice injected with P3 (PDX-P3) displayed high-grade glial proliferation (Fig. 5D) and were strongly positive for nestin (Fig. 5E–G). The tumor biopsies of PDX-P3 indicated a highly elevated Myc and EZH2 gene expression and 4-fold EGFR gene amplification (Fig. 7A). We then assessed HCMV IE1 and UL69 gene presence in the tumor biopsies of PDX-P3. IE1 and UL69 genes were detected in PDX-P3 using qPCR (Fig. 7B–D). We then sequenced the IE1 and UL69 amplicons isolated from PDX-P3 grafted mice. The IE1 and UL69 amplicon sequences in PDX-P3 were mostly analogous to the IE1 and UL69 genes of the HCMV-DB clinical strain (Supplementary Table 4). We detected the presence of IE staining in PDX-P3 tumor biopsies (Fig. 7E). The IE staining was predominantly cytoplasmic, however, some cells presented nuclear staining (Fig. 7F). The IE staining was restricted to the tumor core of the grafted hemisphere and not to the healthy contralateral hemisphere (Fig. 7G). Although IE staining was present only in the tumor core, nestin was detected both in the tumor core and the invasive part (Fig. 7H).

## Discussion

In the present study, we assessed the in vivo transforming capacities of three HCMV strains isolated from GB tumors, namely GB-6638, GB-7720, and GB-9447, as well as HCMV-DB following HAs infection; all the aforementioned strains were previously classified into high-risk transforming strains [17, 24, 36, 37]. In vitro HAs infection with the high-risk HCMV-DB and GB strains resulted in a pro-oncogenic cellular environment with increased Myc and EZH2 expression, sustained growth of CEGBCs, and spheroid formation as well as invasion in 3D cultures. In agreement with EZH2 activation by HCMV, we observed a direct interaction between EZH2 and HCMV lncRNA4.9 transcript, likewise between EZH2 and cellular lncRNA HOTAIR transcript, a poor prognosis oncogenic factor for glioma patients. Upon the stereotactic implantation of the spheroids arising from HAs infected with HCMV-DB and HCMV-GB strains into the brains of Ragγ2C^−/−^ mice, glioblastoma tumors were generated with glial proliferation combining increased cellularity and nuclear atypia with enlarged hyperchromatic nuclei, high nestin expression, EGFR and cMet gene amplification parallel to the detection of IE gene and protein. Interestingly, in the well-known P3-PDX model, high levels of HCMV were detected. Herein, we reported for the first time a proof of concept showing that HAs transformed by HCMV in vitro generate glioblastoma in vivo when engrafted in immunosuppressed mice. Grafts with a glioblastoma-like phenotype originating from HCMV-infected HAs/CEGBCs were termed “CMV-Derived Xenografts” or CDX similar to the previously reported patient-derived xenografts (PDX).

Among eleven HCMV strains isolated from GB tumors, three of them were chosen based on their potential aggressiveness (high GB-6638, intermediate GB-7220 and low GB-9447) to further assess their transforming potential in vivo following the in vitro infection of HAs. After HAs infection, CEGBCs were generated with morphological features matching the previously described CEGBCs-DB and led to the appearance of spheroids with invasion potentials. HCMV-IE1 protein was detected parallel to the stemness marker nestin and the upregulated Myc and EZH2 expression. EZH2 interaction with lncRNA4.9 and HOTAIR transcripts in cultures infected with the three HCMV-GB strains recapitulates the previously observed molecular phenotype induced by HCMV-DB strain. Altogether, the HCMV strains, namely GB-6638, GB-7720, and GB-9447, are present in GB tumors retaining transforming-promoting abilities in vitro, therefore considered as oncogenic strains, and were further tested for their tumorigenicity in vivo.

Among the mechanisms studied to transform HAs and promote disease progression in addition to poor prognosis in GB, is the coupling of Myc and EZH2 overexpression which was observed in our model pointing toward the presence of Myc/EZH2/CEGBCs axis underlying the described results [38, 39]. Accumulated evidence highlights Myc and EZH2 as key players in both oncogenesis and stemness [32, 40]. HCMV lncRNA4.9 interacts with EZH2 [23, 41]. The cellular lncRNA HOTAIR was described to interact with EZH2 in glioblastoma, thus linked to tumor dissemination, proneural-mesenchymal transition (PMT), and drug resistance [32, 33]. The noticeable detection of high lncRNA HOTAIR in the EZH2 IP samples corresponding to GB-6638 explicates the aggressiveness of this particular high-risk HCMV strain, predicting poor prognosis. In contrast, the detection of limited lncRNA HOTAIR interaction with EZH2 in GB-9447 samples supports a better prognosis profile.

High-risk clinical isolates HCMV-DB and HCMV-GB can drive HAs towards oncogenic transformation in vitro with the appearance of CEGBCs in chronically infected cultures that possess a glioblastoma-like phenotype [9]. Stemness acquisition, commonly described in metastasis and poorly differentiated tumors [42, 43], is in accordance with the previous findings where GB-generated spheroids are composed of glioma stem cells. The concomitant presence of the stemness marker nestin and HCMV-IE1 was detected in the spheroid structures generated from CEGBCs, as reported for nestin in the cell lines derived from GB [44]. Interestingly, the concomitant presence of viral proteins and nestin within transformed cells has been reported for the two herpes oncoviruses, namely Epstein-Barr virus and Kaposi sarcoma herpes virus [45, 46].

To test the relevance of HCMV transformation of HAs in vivo, we developed an orthotopic CMV strain-Derived Xenograft (CDX) similar to the patient-derived xenograft (PDX) usually used to study glioblastoma pathogenesis and therapeutic intervention [34]. Therefore, we assessed in parallel the generation of glioblastoma tumors in mice engrafted with CEGBCs spheroids obtained following the infection of HAs with the three HCMV-GB strains as well as HCMV-DB. We used P3 cells PDX as a positive control [29]. A breakpoint in the mice’s weight was observed earlier at day 40 for CDX-6648 and CDX-7220 post-engraftment and at day 50 for CDX-9447 indicating a potential generation of glioblastoma tumors in vivo with a more aggressive phenotype in CDX-6648 and −7220 compared to CDX-9447. Using HE staining, glial proliferation combining increased cellularity and nuclear atypia with enlarged hyperchromatic nuclei was present in the four CDX (6638, 7220, 9447, DB) and was more pronounced in PDX-P3. This was further confirmed by IHC showing a high nestin expression in CDX-6648 and CDX-7220 compared to CDX-9447. In addition to nestin expression, the detection of GFAP staining could be related to reactive astrocytes which are well-known as the result of cellular stress following viral infection thereby favoring tumor aggressiveness [47, 48]. Interestingly, the highest nestin level was detected in CDX-6648, compared to the intermediate level in CDX-7220 and the low level detected in CDX-9447 (Fig. 5G). EGFR and cMETgene amplifications, linked to invasiveness and poor prognosis respectively [49, 50], were mostly observed in CDX-6648 and CDX-7220 compared to CDX-9447 (Fig. 5H). Interestingly, the HCMV-GB strains 6648 and 7220 that possess the more aggressive phenotype in CDX were isolated from glioblastoma with unmethylated MGMT promoter, meanwhile, HCMV-GB strain 9447 with a less aggressive phenotype in CDX was isolated from a glioblastoma patient with methylated MGMT promoter (Suppl. Table 3). Notably, even after 2 months post-engraftment we were able to detect the HCMV gene and protein in CDX further indicating a potential link between HCMV presence and progression in the tumor. The tumor microenvironment with enhanced Myc and EZH2 gene expression observed in CDX was similar to the one observed in the biopsies of GB patients, further indicating the clinical relevance of our animal model. Finally, we observed that the PDX-P3 model had similar yet more marked HES and nestin-IE phenotypes compared to the GB-CDX models, thus questioning the role of HCMV in GB initiation and progression. It is of relevance that specific immune-cells recognize CMV-infected cells such as γδ T cells. These are an adaptive small T cell subset based on immunological ontogeny retaining both innate and adaptive characteristics [51]. Thus, it would be interesting to investigate, in a follow-up project, the effect of γδ T cells on GB development since they could represent a new approach to therapeutic intervention.

Recently, specialized humanized murine models tailored for HCMV have been created; in these models, mice are implanted with human cells or tissues which support HCMV infection [52, 53]. Though mouse models containing exclusively human fetal tissue xenografts can be infected with HCMV, they lack the capability to address inquiries regarding latency, persistence, and dissemination. Recognizing that HCMV latency, reactivation, and ensuing disease occur within the framework of functional immune responses, a humanized mouse model was generated. This model involves the reconstitution of mice with a bone marrow transplant equivalent, along with matched human fetal liver and thymus tissue [54]. Advancements in humanized mouse models incorporating a stronger human immune system and the engraftment of other cell types beyond the hematopoietic lineage, such as epithelial cells and human astrocytes, could pave the way for new avenues of investigation. This may contribute to a deeper understanding of the HCMV-host relationship.

Based on the 2021 WHO classification of tumors of the central nervous system, both histologic features and genetic alterations are incorporated into the diagnostic framework, classifying and grading brain tumors [2]. Thus, the presence of any of the following five criteria is sufficient to designate an IDH-wildtype diffuse astrocytic glioma as glioblastoma: microvascular proliferation, necrosis, TERT promotor mutation, EGFR gene amplification, or 17/–10 chromosome copy number changes [2]. We observed diffuse astrocytic/glial proliferation with marked EGFR gene amplification in the four CDX (6638, 7220, 9447, DB) and PDX-P3, in line with the generation of glioblastoma in mice engrafted with human cytomegalovirus-infected astrocytes.

While the current scientific consensus attributes the development of glioblastoma to genetic mutations and several risk factors, some emerging research suggests a potential association between certain viruses and glioblastoma. HPV and EBV infections have been associated with GB [55, 56]. Thus, it is worth mentioning that the GB biopsies used in our study exclusively indicated the presence of HCMV where neither HPV nor EBV were detected in the samples.

In conclusion, the CDX model described in the present study could highlight the molecular mechanisms used by HCMV to transform astrocytes and lead to tumor initiation in vivo and tumor invasion and progression comparable to well-established patient-derived GB cell lines. Furthermore, it represents an ultimately valuable model, in addition to the well-known PDX model, for studying physiopathology and therapeutic intervention for glioblastoma treatment. Anti-HCMV therapy and immunotherapy seem to be of particular interest in this context.

### Supplementary information

Supplementary data

## Data Availability

The data supporting the findings of this study are available within the article and its Supplementary Information files and from the corresponding authors on request.
