# Supplementary material for: Generation of glioblastoma in mice engrafted with human cytomegalovirus-infected astrocytes
Source: Cancer Gene Ther. 2024 Mar 29;31(7):1070–80. doi: 10.1038/s41417-024-00767-7 (PMC11257955; doi:10.1038/s41417-024-00767-7)
Supplement: Supplementary file 1 — Supplementary data [file 41417_2024_767_MOESM1_ESM.docx]

**Supplementary Data**

# Supplementary Materials and Methods

## Cell cultures

Primary human astrocytes (HAs) were purchased from Innoprot (Derio, Spain) and cultivated in astrocytes medium (Innoprot). They were cultured under standard conditions (37°C, 5% CO_2_, 95% humidity) and verified as mycoplasma-free as determined by monthly screenings (VenorGem classic mycoplasma detection, Minerva Biolabs).

## Viral growth and detection

HAs (1x10^6^) cells infection with the HCMV clinical isolates was performed at a multiplicity of infection (MOI) of 1. For HCMV quantification, cell-free infectious supernatant was collected, DNA was isolated (EZNA Blood DNA Kit, D3392-02, Omega BIO-TEK, Norcross, GA) and real-time quantitative PCR (qPCR) was performed using a Stratagene Mx3005P thermocycler (Agilent Technologies, Santa Clara, CA) and IE1 as well as UL69 primers. qPCR was carried out using KAPA SYBR FAST Master Mix (KAPA BIOSYSTEMS, Potters Bar, UK). Results collection and analysis were done using MxPro qPCR software. Primers used are listed in Supplementary Table 1.

## Isolation and growth of CEGBCs

CEGBCs were initially cultivated in HAs medium complemented with low levels of fetal bovine serum (2%) as per the manufacturer’s recommendations. Since CEGBCs resemble stem cells compared to uninfected HAs, serum was excluded at day 5 post-infection and this is to fit with the optimal conditions of serum-free stemness growth as requested for glioblastoma cell cultures [1]. Olympus optical microscope (Olympus Corporation, Tokyo, Japan) and OPTIKA digital camera (Optica Microscopy, Ponteranica, Italy) were used to monitor the long-term cultures of infected astrocytes and CEGBCs.

## Reverse transcriptase quantitative polymerase chain reaction (RT-qPCR)

The detection of transcripts was assessed by RT-qPCR as detailed previously [2]. Briefly, total RNA was extracted using an RNA extraction kit (EZNATotal kit I,Omega BIO-TEK), and reverse transcription was performed using the SuperScript IV First-Strand Synthesis kit (Invitrogen, Carlsbad, CA, USA). The expression of markers was measured by real-time qPCR using a KAPA SYBR FAST Master Mix (KAPA BIOSYSTEMS, KK4601) and specific primers according to the manufacturer’s protocol. The fold change expression was calculated using delta-delta Ct method for biopsies samples and ratio method for CDX samples. Primers used are listed in Supplementary Table 1.

## Real-time quantitative PCR (qPCR) assay

Genomic DNA was isolated from CDX sample using EZNA Blood DNA Kit (D3392-02, Omega BIO-TEK, Norcross, GA). IE1, UL69, EGFR, c-Met and chromosome 17 was assessed by real-time qPCR using a KAPA SYBR FAST Master Mix (KAPA BIOSYSTEMS, KK4601) and specific primers according to the manufacturer’s protocol. Primers used are listed in Supplementary Table 1.

## RNA cross-linking immunoprecipitation (RNA CLIP) assay

RNA CLIP assay was performed on uninfected HAs and infected HAs as previously reported [3]. qPCR analysis of EZH2 immunoprecipitated samples (IP EZH2) and negative control (IP IgG) were normalized with respect to each input and expressed as (2(−ΔCt)) x 100 (% Input) as previously reported [4]. lncRNA4.9, lncRNA HOTAIR primers sequences are listed in Supplementary Table 1.

## Spheroid formation assay

Single cells (1x10^4^) isolated by accutase were seeded in a serum-free astrocytes medium containing methylcellulose.

## Invasion Assays

Collagen invasion assay: Collagen I (Corning, New York, NY) of 1 mg/ml concentration was prepared in 1X PBS with 7.2mM NaOH and 0.1% HCl was added [5,6]. Prepared spheroids were incubated on ice for 30 min and then separately selected, washed in PBS, and subsequently included in the collagen solution. After 1 hour at 37°C in a cell incubator, serum-free astrocytes medium was added.

## Statistical analysis

Quantitative results are reported as mean ± SD of independent experiments. Statistical analyses were done using Wilcoxon-Mann-Whitney test; a p-value≤0.05 was considered to be statistically significant [*: ≤0.05; **: ≤0.01; ***: ≤0.001]. Microsoft Excel with Inkscape was used to construct the plots and histogram data.

**References:**

[1] Hong X, Chedid K, Kalkanis SN. Glioblastoma cell line-derived spheres in serum-containing medium versus serum-free medium: A comparison of cancer stem cell properties. International Journal of Oncology 2012;41:1693–700. https://doi.org/10.3892/ijo.2012.1592.

[2] Haidar Ahmad S, Pasquereau S, El Baba R, Nehme Z, Lewandowski C, Herbein G. Distinct Oncogenic Transcriptomes in Human Mammary Epithelial Cells Infected With Cytomegalovirus. Front Immunol 2021;12:772160. https://doi.org/10.3389/fimmu.2021.772160.

[3] Rossetto CC, Tarrant-Elorza M, Pari GS. Cis and Trans Acting Factors Involved in Human Cytomegalovirus Experimental and Natural Latent Infection of CD14 (+) Monocytes and CD34 (+) Cells. PLoS Pathog 2013;9:e1003366. https://doi.org/10.1371/journal.ppat.1003366.

[4] Battistelli C, Cicchini C, Santangelo L, Tramontano A, Grassi L, Gonzalez FJ, et al. The Snail repressor recruits EZH2 to specific genomic sites through the enrollment of the lncRNA HOTAIR in epithelial-to-mesenchymal transition. Oncogene 2017;36:942–55. https://doi.org/10.1038/onc.2016.260.

[5] Daubon T, Guyon J, Raymond A-A, Dartigues B, Rudewicz J, Ezzoukhry Z, et al. The invasive proteome of glioblastoma revealed by laser-capture microdissection. Neuro-Oncology Advances 2019;1:vdz029. https://doi.org/10.1093/noajnl/vdz029.

[6] Guyon J, Andrique L, Pujol N, Røsland GV, Recher G, Bikfalvi A, et al. A 3D Spheroid Model for Glioblastoma. JoVE 2020:60998. https://doi.org/10.3791/60998.

# Supplementary Tables

**Supplementary Table 1. List of primers used.**

| **Primer** | **Primer Sequence** |
| --- | --- |
| IE1-forward | 5'-CGACGTTCCTGCAGACTATG-3' |
| IE1-reverse | 5'-TCCTCGGTCACTTGTTCAAA-3' |
| UL69-forward | 5’-GGGATGTCGATGACTCCCTTC-3’ |
| UL69-reverse | 5’-GTCGCTATTGGATCTCACCGT-3’ |
| EZH2-forward | 5’-TCGTGCCCTTGTGTGATAGC-3’ |
| EZH2-reverse | 5’-TCTCGGACAGCCAGGTAGC-3’ |
| Myc-forward | 5’-ACACCCTTCTCCCTTCG-3’ |
| Myc-reverse | 5’CCGCTCCACATACAGTCC3’ |
| LncRNA4.9-forward | 5’-GTGAACCGATACGGGTGGAG-3’ |
| LncRNA4.9-reverse | 5’-CATTTGAACAGAGAAAGGTGG-3’ |
| LncRNA HOTAIR-forward | 5’-GGTAGAAAAAGCAACCACGAAGC-3’ |
| LncRNA HOTAIR-reverse | 5’-ACATAAACCTCTGTCTGTGAGTGCC-3’ |
| c-MET-forward | 5’-CATCTCAGAACGGTTCATGCC-3’ |
| c-MET-reverse | 5’- TGCACAATCAGGCTACTGGG-3’ |
| EGFR-forward | 5’-TGCGTCTCTTGCCGGAAT-3’ |
| EGFR-reverse | 5’- GGCTCACCCTCCAGAAGGTT-3’ |
| GAPDH-forward | 5’-CCCCTCTTCAAGGCCTCTAC-3’ |
| GAPDH-reverse | 5’-CGACCACTTTGTCAAGCTCA-3’ |
| Akt-forward | 5’-ATCCCCTCAACAACTTCTCAGT-3’ |
| Akt-reverse | 5’-CTTCCGTCCACTCTTCTCTTTC-3’ |
| Chr17_1a- forward | 5' GGGATAATTTCAGCTGACTAAACAG 3' |
| Chr17_2b-reverse | 5' TTCCGTTTAGTTAGGTGCAGTTATC 3' |
| Epstein-Barr virus (EBV)-forward | 5'-GATTTGGACCCGAAATCTGAT-3' |
| Epstein-Barr virus (EBV)-reverse | 5'-TCTGGGGGCTTATTCCTCTT-3' |
| Human papillomavirus (HPV) 16-E6-forward | 5’-GCACCAAAAGAGAACTGCAATGTT-3’ |
| Human papillomavirus (HPV) 16-E6-reverse | 5’-AGTCATATACCTCACGTCGCAGTA-3’ |

**Supplementary Table 2. List of antibodies used.**

| **Antibody** | **Catalog Number/Source** |
| --- | --- |
| Anti-Myc Tag | 06-549-25UG/Merck KGaA, (Darmstadt, Germany) |
| EZH2 | AB_2793397/Active Motif (Carlsbad, CA, USA) |
| CMV pp72 (IE1) | SC-69834/Santa Cruz Biotechnology (CA, USA) |
| IE1/2 | ab53495/Abcam (Cambridge, UK) |
| Nestin | SC-23927/Santa Cruz Biotechnology (CA, USA)  PA5-11887/ ThermoFisher (MA, USA) |
| GFAP | GFAP #3670/Cell Signalling (MA, USA) |
| PE-conjugated anti-mouse antibody | BD-551436/BD Biosciences (Franklin Lakes, USA) |
| FITC-conjugated anti-rabbit antibody | ab6717/Abcam (Cambridge, UK) |
| FITC-conjugated Rat Anti-Mouse | BD-553443/BD Biosciences (Franklin Lakes, USA) |
| TRITC-Goat Anti-Rabbit IgG | ab6718/Abcam (Cambridge, UK) |

**Supplementary Table 3. GB patients tested for CDX.**

| Patient | Age | Sex | M/UM |
| --- | --- | --- | --- |
| **6638** | 65 | M | UMETH |
| **7220** | 50 | M | UMETH |
| **9447** | 69 | F | METH |

**Supplementary Table 4. Analysis of IE1 and UL69 amplicon sequences detected in grafted mice brains.**

| **Gene** | **Samples** | **Identity to HCMV-DB Genome** | **Position of identities** |
| --- | --- | --- | --- |
| **IE1** | CDX-6638 | 100% | 173583-173700 |
|  | CDX-7220 | 100% | 173583-173700 |
|  | CDX-9447 | 99% | 173583-173700 |
|  | CDX-DB | 97% | 173608-173684 |
|  | PDX-P3 | 100% | 173583-173700 |
| **UL69** | CDX-6638 | 98% | 99856- 100048 |
|  | CDX-7220 | 98% | 99850- 100048 |
|  | CDX-9447 | 98% | 99850- 100044 |
|  | CDX-DB | 100% | 99851- 100048 |
|  | PDX-P3 | 98% | 99880- 100048 |

# Supplementary Figures


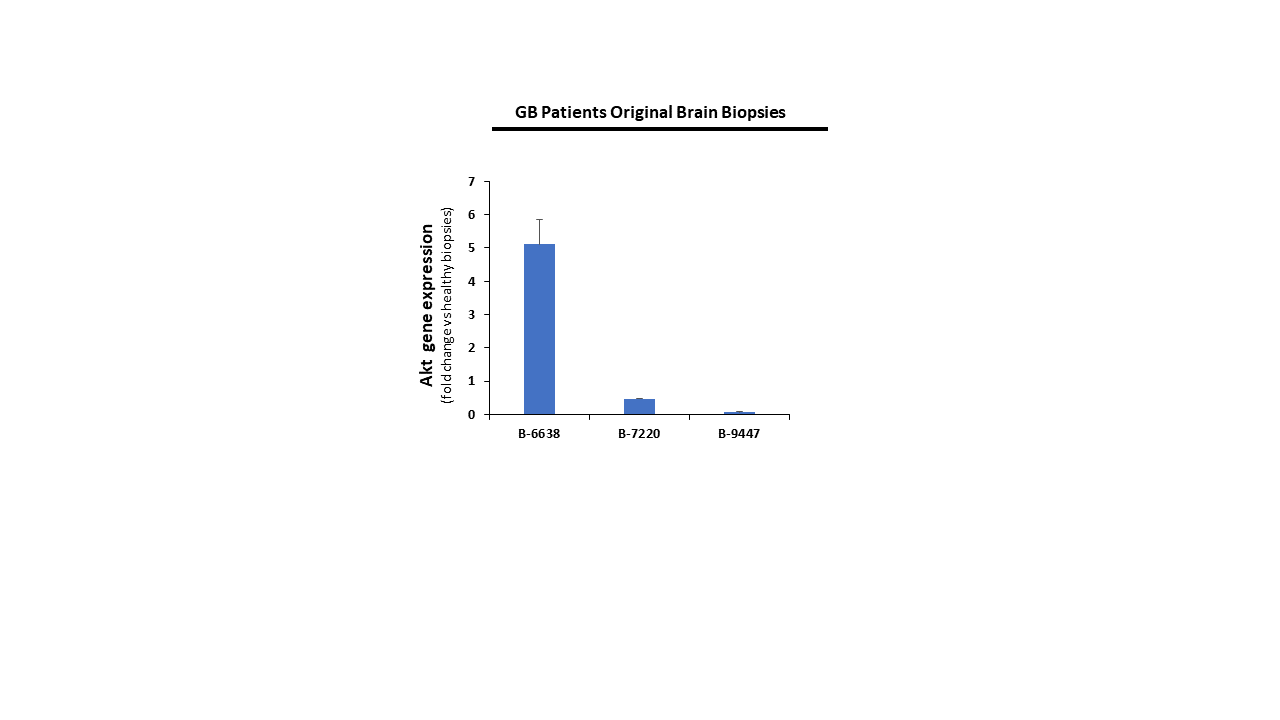


**Supplementary Figure 1. Tumor landscape of the three oncogenic HCMV strains isolated from GB patients based on Akt gene expression as measured by RT-PCR.**
